# ‘It's Primarily Around Their Viral Load’: Public Health Decision‐Making and HIV Risk Assemblages in Ontario, Canada

**DOI:** 10.1111/1467-9566.70220

**Published:** 2026-06-22

**Authors:** Emerich Daroya, Martin French, Colin Hastings, Andrea Krüsi, Stephen Molldrem, Maureen Owino, Ryan Peck, Amy Wah, Alexander McClelland

**Affiliations:** ^1^ Department of Politics, Media and Philosophy La Trobe University Melbourne Victoria Australia; ^2^ Dalla Lana School of Public Health University of Toronto Toronto Ontario Canada; ^3^ Department of Sociology and Anthropology Concordia University Montreal Quebec Canada; ^4^ Department of Sociology and Legal Studies University of Waterloo Waterloo Ontario Canada; ^5^ School of Criminology Simon Fraser University Burnaby British Columbia Canada; ^6^ School of Public Health and Population Health The University of Texas Medical Branch Galveston Texas USA; ^7^ Faculty of Environment and Urban Change York University Toronto Ontario Canada; ^8^ HIV & AIDS Legal Clinic Ontario Toronto Ontario Canada; ^9^ Institute of Criminology and Criminal Justice, Carleton University Ottawa Ontario Canada

## Abstract

In Ontario, Canada, public health authorities can issue and enforce orders to people living with HIV (PLWH). However, how public health practitioners determine when someone's behaviours constitute ‘significant’ risk remains underexplored. We drew on assemblage theory to examine how HIV risk is co‐constituted through the interplay among biomedical technologies, institutional practices, legal frameworks and social discourses. We conducted 18 semi‐structured interviews with public health personnel and used reflexive thematic analysis. Five interrelated themes emerged. First, high viral load was considered a potential risk indicator when associated with high‐risk activities. Second, discontinuity in HIV care may flag individuals for intervention. Third, co‐infection with sexually transmissible and blood‐borne infections (STBBI) triggered a viral load review to assess HIV exposure potential. Fourth, noncompliance with public health directives positioned PLWH as needing management. Fifth, Undetectable = Untransmittable (U = U) discourses have reconfigured and neutralised risk, functioning as a technology of reflexive governance. However, uptake of U = U was described as uneven across Ontario's public health units, leading to variable, and sometimes coercive, approaches. By conceptualising HIV risk as emergent, this study challenges individualised notions of responsibility and highlights the relational production of risk. Adoption of U = U and equity‐based public health practices is needed to ensure nonpunitive HIV responses.

## 1 Introduction

Public health organisations and authorities have been responsible for addressing the HIV/AIDS epidemic since the outbreak started across many parts of the world. Public health has been tasked with identifying and managing populations considered to pose a risk to community health. From the beginning of the outbreak, people living with HIV (PLWH) emerged as ‘risky persons’, considered infectious and dangerous, and subjected to surveillance and control (Burris and Gostin 2007). In Canada, public health institutions have used coercive measures against individuals considered risks to community health (Klein 2009). Public health laws have been enacted to manage HIV, including mandatory reporting and contact tracing (Flanagan 1988; Klein 2009; Lunny and Shearer 2011). Canada's healthcare model, where the federal government provides funding whereas provinces and territories hold responsibility for service delivery, has shaped how public health has addressed HIV. Ontario has been recognised as having enforced the country's most stringent mandatory contact tracing programme (Flanagan 1988; Kinsman 1996; O’Byrne and Bryan 2013). In Ontario, HIV is a reportable infection, and laboratories must report all positive HIV results to public health units (PHUs). Providers are also required to report persons suspected or confirmed to have HIV from nominal test results, and patients may be compelled to cooperate with contact tracing by public health authorities. However, when anonymous testing is used, public health's ability to exercise these powers is limited because no identifying information is reported (HIV Testing Ontario 2020). Although providers and patients may assist with notification, PHUs conduct the majority of contact tracing for HIV and other sexually transmissible and blood‐borne infections (STBBIs) (Ontario Ministry of Health and Long‐Term Care 2009). PHUs in Ontario have even issued orders to index cases to force them to name contacts or require contacts to present for assessment (Ontario Ministry of Health and Long‐Term Care 2009).

Medical health officers in Ontario can issue these orders under Section 22 of the Health Protection and Promotion Act (HPPA) (O’Byrne and Bryan 2013). Due to HIV's designation as a communicable disease, medical officers of health are authorised under HPPA's Section 22 to order people to take measures to reduce or eliminate transmission risk. This includes coercing PLWH to disclose or avoid engaging in sexual activities that could expose someone to HIV risk (e.g., condomless sex). Notably, the law does not require a sunset clause (i.e., limiting the time frame of the order), and failure to comply can result in a fine of up to $5000 per day, which can also be turned into court orders, the breach of which could lead to jail time (O’Byrne and Bryan 2013). Between 1985 and 1993, medical health officers in Ontario issued 45 written orders to PLWH, increasing to 55 between 1999 and 2003 (Grant 2008). No publicly available data have been reported since, despite qualitative research indicating that Section 22 orders continue to be part of the public health response to HIV (Mykhalovskiy 2011). Additionally, PLWH have been found to distrust case managers, believing that public health authorities can exercise coercive and punitive powers against them (Orser and O’Byrne 2021).

PLWH's distrust in public health is tied to a lack of clarity about how public health authorities determine when a PLWH's behaviours are considered ‘risky’ or threatening to public health, and what actions they take thereafter. Previous research has suggested that the designation of risk usually involves the discretionary judgement of medical officers and public health authorities based on public health laws in their jurisdictions (Berger 1990). Although there have been studies regarding the impacts of Section 22 orders and the effects of HIV criminalisation on PLWH and public health practice in Canada (e.g., Hastings et al. 2024; McClelland 2019, 2024; Mykhalovskiy 2011), there is currently limited research examining how public health authorities determine when behaviours by PLWH constitute significant risk to warrant coercive interventions. We aimed to investigate how PLWH are constituted and emerge as risks through public health institutional and personnel practices.

### 1.1 Public Health and HIV Risk Assemblages

Public health authorities and organisations are generally focused on managing and improving population health. Public health comprises professionals, primarily medical and health workers in state‐sponsored organisations such as PHUs, responsible for the ‘regulation and surveillance of individual bodies and the social body as a whole’ to eliminate risks (Peterson and Lupton 1997, 3). For Lupton (1995), public health risks generally fall into three categories. First, there are external risks to the population, such as environmental hazards. Second, there are internal risks, purported to be consequences of individual ‘lifestyle’ choices, such as smoking or alcohol consumption. To manage threats, public health encourages individuals to evaluate risks and change behaviours accordingly. Lastly, some individuals or groups are considered ‘at risk’ because they are vulnerable to developing health issues or exposure to communicable diseases. At‐risk individuals and groups are targets of public health surveillance to prevent the acquisition and transmission of communicable diseases.

These risk categories are also central to the management of HIV. PLWH are often positioned by public health as external transmission risks to others due to perceived ‘deviant’ behaviours, such as injecting drugs or having sex without prophylaxis use (Lupton 2013). Those who acquire HIV are usually blamed for engaging in ‘risky’ behaviours and failing to manage risks, and those who are labelled ‘at risk’ become targeted for prevention messaging. To be labelled as an at‐risk group can have a range of consequences. On the one hand, some groups considered at risk can use their labelling to acquire resources for prevention programming. On the other hand, it could reinforce ideas of blame, threat and stigmatisation, which can even lead to the use of public health interventions and criminalisation, especially for vulnerable groups, including PLWH who are racialised, 2S/LGBTQ+ (Two‐Spirit, lesbian, gay, bisexual, trans and queer) and/or sex workers (Owczarzak 2009).

Notions of risk are prone to change. As Lupton writes, ‘[e]pidemiological and medical definitions of what behaviours constitute risky behaviours, and how these behaviours in turn affect health status, are subject to continual change’ (Lupton 1995, 83). Biomedical advances in the management and prevention of HIV have significantly shifted how HIV risk is viewed. For example, with the scientific consensus surrounding undetectable = untransmittable (U = U), many public health authorities no longer consider a PLWH who adheres to antiretroviral therapy (ART) and maintains a viral load of less than 200 copies/mL measured every 4–6 months as an HIV risk, for they cannot sexually transmit HIV (Public Health Agency of Canada 2023). Additionally, the availability of HIV pre‐exposure prophylaxis (PrEP) and post‐exposure prophylaxis (PEP) for people who do not have HIV can significantly reduce their risk of acquiring the virus.

The idea of HIV risk evolving through and with different biomedical technologies resonates well with the conceptualisation of risk from what has been termed a ‘more‐than‐human’ perspective (Lupton 2023). This suggests that materialities and relationships between humans and nonhumans shape experiences, identities, subjectivities and even notions of risk (Lupton 2023). One of the most prominent theories within this framework is that of assemblages, as developed by Deleuze and Guattari (1987). In relation to risk, Lupton (2023) suggests that risks are ‘actively made’ and ‘brought into being as risk assemblages’ through the ‘gathering of various agents and actors [humans or otherwise], and how seemingly separate risks are related to each other through dense networks of connection’ (Lupton 2023, 37). Assemblages are constantly changing as different configurations of human and nonhuman forces come together in a continuous cycle of production and change (Alldred and Fox 2015; Daroya 2022; Lupton and Lewis 2022; Lupton 2023). From this perspective, risk assemblages are ‘always in the process of becoming’ (Lupton 2013, 638). This means that risks are constantly shifting and co‐constituted relationally in practice (Odger et al. 2019). In this article, we deploy assemblage frameworks to rethink HIV risk as dynamic, relational and emergent through changing biomedical, public health and legal approaches to HIV, rather than considering it as static and inherent to individuals.

Several scholars have deployed assemblage thinking to study the effects of HIV biomedical technologies on sexualities, subjectivities and risk (Rosengarten and Michael 2009; Guta and Newman 2021; Brown and Di Feliciantonio 2022; Daroya 2022). Drawing on Deleuzoguattarian assemblages, Guta and Newman (2021) suggest that viral load measurements have become a significant component of what it means to be at risk or to be living with HIV. Being diagnosed with HIV entails becoming enrolled into a care continuum with clearly defined goals and outcomes for treatment initiation, adherence, viral suppression and continuing engagement in care (Guta et al. 2016; Guta and Newman 2021). However, more than changing the dynamics of care, biomedical treatments have also created a complex assemblage intertwined with notions of responsibility, public health authorities and legal forces (Guta and Newman 2021). Consistent engagement with HIV care has become a measure of personal responsibility. Viral load measurement has shifted from a clinical diagnostic measure to a reportable data point, allowing public health authorities to determine whether someone is not virally suppressed or in care (Guta and Newman 2021). In the Canadian context, as Guta and Newman describe, ‘persons charged with HIV nondisclosure have had their viral load publicly reported by police through media and in one instance an individual received court‐mandated treatment orders for not remaining adherent to their medication’ (Guta and Newman 2021, 1523). This means that viral load measurement is an important component of HIV risk assemblages, contributing to the determination of PLWH as risks and has profound legal and criminal implications that can be used by public health authorities to enact coercive and punitive interventions.

Drawing on assemblages is particularly useful for understanding how notions of HIV risk are co‐constituted and emerge through complex connections between PLWH, public health personnel, viral load, public health legislation, the care continuum and ART, among others. A more‐than‐human perspective enables us to theorise how HIV risk is not fixed but constantly reconfigured. Analysing qualitative interview data with public health practitioners in Ontario, we describe how notions of risk emerge through assemblages of biomedical technologies, legal frameworks, institutional practices and other human and nonhuman forces. Importantly, we demonstrate how viral load measurement operates as a *technology of reflexive governance*, used to determine or even neutralise risk.

## 2 Methods

Our analysis draws on data from *Mapping Sero Surveillance,* qualitative study involving interviews with public health personnel, clinicians and PLWH who have experience interacting with public health in Ontario, Canada. The study had four overarching and interrelated objectives. First, to map how blood and information collected from people living with HIV moved from clinical to public health settings. Second, to understand how and why people living with HIV were considered as risks by public health authorities. Third, to build the capacity of communities living with and affected by HIV to understand public health intervention and surveillance practices. Finally, to realise public health approaches that prioritise human rights, ethics, trust, equity and bodily autonomy. This paper addresses the second objective by analysing interviews with public health personnel. Although interviews were also conducted with PLWH, they focused mainly on lived experiences and, although valuable, including these data would significantly diverge from the objectives of this paper. The study was approved by the Research Ethics Boards of Carleton University (#115809), Concordia University (#30018509), the University of Waterloo (#45146), the University of British Columbia (#H21‐02496‐A005) and the University of Toronto (#44572).

Recruitment of participants leveraged our network and direct contact with public health authorities across Ontario using publicly available contact information. Recruitment involved purposive and respondent‐driven snowball sampling, with participants asked to refer colleagues who might be interested. To ensure we garnered a diverse cross‐section of both policy and practice, we sought a range of expertise and institutional locations, including individuals engaged in policy and surveillance, nurses, physicians, laboratory technicians, administrators and researchers. We prioritised recruiting public health personnel known for working with racialised communities, women, sex workers, 2S/LGBTQ+ people, people who use drugs, migrants, refugees and Indigenous communities, as they are likely to have navigated HIV risk and marginalisation in practice.

Between March 2023 and January 2024, we conducted semi‐structured interviews with 18 public health personnel across Ontario. Semi‐structured interviews allowed participants to collaborate on data construction, supporting the rigour and relevance of the data collected. All participants provided written consent before interviews, which lasted between 60 and 90 min and were conducted online. We asked participants to speak about their day‐to‐day work and the steps they follow to conduct a new HIV intake, including how they convey information about HIV and the law. As public health officials are mandated to monitor behavioural compliance, we further probed participants to describe the kinds of information they use to determine when someone poses a potential risk and which policies, practices and processes are involved. Interviews were digitally recorded and transcribed verbatim using an AI software (Otter.ai). To protect anonymity and confidentiality, participant interviews were de‐identified and each participant was provided with a pseudonym chosen specifically for this paper. Identifying details, including clinic names, public health unit locations, job titles, cities and towns, the names of internal data management systems, along with unique identifying speech patterns, were redacted and/or replaced with generic names. Protection of both anonymity and confidentiality was paramount to ensure participants could speak honestly without concerns to their professional practice.

Using a participatory analysis approach, we employed a joint team analysis process to collaborate on data interpretation, bringing together our larger interdisciplinary team of researchers from different backgrounds, including sociology, law, public health, criminology, health equity, community‐based organisations and PLWH (Cornish et al. 2014; McClelland and Bruckert 2022). This joint process was intended to bring diversity of perspectives to the analysis and lay out a systematic approach for multiple coders to ensure accountability and collaboration. We coded the transcripts deductively using Dedoose qualitative data management software, using line‐by‐line coding, focussing on what we expected to see in the transcripts (Bradley et al. 2007). Data analysis followed a multi‐stage iterative process, drawing on inductive and deductive approaches. We first coded the transcripts deductively using Dedoose qualitative data management software. The initial coding framework was developed based on key themes reflected in the interview guide, observations made upon reviewing the transcripts, and known topics/concepts in relevant literature (Bradley et al. 2007). Data were subjected to a process in which we identified categories, summarised the content of each category and examined contradictory evidence.

We then developed coding through a series of subgroup meetings held in 2024 to deepen our inductive analysis. In these meetings, we enhanced coding frameworks by testing the boundaries of categories. We further refined the analysis by using smaller teams to elucidate themes generated from the interviews, informed by reflexive thematic analysis (Fereday and Muir‐Cochrane 2006; Braun and Clarke 2021). In reflexive thematic analysis, the themes generated do not exist separately from the researchers but are mediated by the values, skills and training they bring to the analytic process (Braun and Clarke 2021). Throughout the analysis, our team drew on our multiple positions as HIV researchers and advocates. This reflexivity included our knowledge about HIV criminalisation, stigmatisation and marginalisation, including lived experiences.

Analysis was informed by the assemblages framework outlined above, focussing on how PLWH were co‐constituted as risks by public health authorities through a complex assemblage. This approach meant focussing on the interplay between public health actors and PLWH, legal frameworks, public health policies, biomedical knowledge and the interpretation of laws and policies in practice. Below, we identify the human and nonhuman relations that assemble around HIV risk, the different capacities and effects they produce and how HIV risk is connected to legal and institutional frameworks.

## 3 Results

Five interrelated forces involving PLWH, public health personnel, clinics, medical technologies and legal apparatuses emerged in our analysis, co‐constructing whether a person and their behaviours constitute HIV transmission risk. First, many participants described high viral load, accompanied by potential HIV transmission behaviours, as a significant risk indicator. Second, public health practitioners alluded to the importance of care infrastructure, particularly care linkage and medication adherence, as key factors in the shaping of risk. Third, STBBI co‐infection may be used to assess risk, but only when it is associated with a high viral load. Fourth, public health orders, specifically noncompliance with directives, may be used to designate risk. However, fifth, participants noted the significance of U = U discourses, which have reconfigured approaches to risk. That is, if someone has an undetectable viral load, public health authorities do not consider them as risky. Therefore, viral load is a significant force, emerging as a technology of reflexive governance and used to determine or even neutralise risk. Drawing on the assemblages framework, our analysis revealed that HIV risk is not static or intrinsic to individuals; instead, it is processual and emergent.

### 3.1 High Viral Load

Many participants spoke about viral load as an important force in HIV risk assemblages. Viral load is not simply a biomedical status; it also serves as an indicator of adherence to antiretroviral medications and, most importantly, actively constructs risk and infectiousness. For example, a participant explained:

It’s primarily around their viral load […]. So, if they have a detectable viral load, they’re kind of above 200. They can potentially transmit to other individuals.

(Casey, laboratory scientist)

This excerpt demonstrates how a detectable viral load, confirmed through laboratory tests, makes someone a risk. The use of viral load highlights how HIV risk emerges through a complex assemblage of bodies, blood samples, laboratory scientists and their equipment, medical health officers and legislation, among others. One participant further shared:

So it has been brought to my attention for individuals on section orders that were issued in the past […] or in other jurisdictions that this individual now has a viral load or an increase […] or change in their status, which could, in theory, mean that there’s a higher risk of exposure […]. But, you know, when you see these lab results, sometimes this raises the concern of public health nurses.

(Jessie, public health policy/surveillance)

Viral load travels across different networks—from individuals to laboratories to electronic medical records accessed by PHUs. Each element in the network plays a vital role in shaping risk, which, in turn, informs potentially coercive public health interventions. Together, the relational connections among viral load information, public health legislation and personnel are drawn to determine risk. It must be noted, however, that a high viral load does not automatically constitute a PLWH as a risk. To be considered as such requires other elements to be drawn into the HIV risk assemblage, including behaviours that may expose others to HIV. One participant explained:

You’d have to have reasonable evidence that there’s ongoing concern regarding transmission. So that might be a time that you would consider a client’s viral load.

(Parker, physician/administrator)

This statement illustrates that whereas a high viral load is a significant factor, other elements, such as transmission potential, are also considered and incorporated into the HIV risk assemblage. Further, these comments suggest that some public health personnel reflexively interpret what HIV risk means in the context of U = U.

In addition to viral load and transmission risk, activities that could potentially increase HIV exposure, such as engaging in sexual intercourse or sharing injection equipment, are also considered. For example, one participant shared:

So, there’s kind of […] two components. One is viral load. So, if someone has a high viral load, that has to be joined also with activity that could transmit disease […]. If someone has a high viral load and isn’t having sex with anyone and not sharing any needles, we don’t really care.

(Willow, public health nurse)

Willow's response suggests that even though PLWH engage in practices that could be perceived as ‘risky,’ having an undetectable viral load significantly minimises HIV risk and, therefore, further actions from public health authorities are not warranted. This implies that public health experts draw on multiple factors to assess risk, and that a single factor, such as risk‐related activities, does not definitively make PLWH risky. Instead, other variables, especially viral load, are considered.

Additionally, participants described HIV nondisclosure as another indicator of risk, along with a detectable viral load. Another participant explained:

So, a healthcare provider or organisation might call us and say, you know, I have this client who’s indicated that they’re unwilling to disclose their HIV status, they’re not virally suppressed, they’re not in care.

(River, public health nurse)

Information about potentially risky behaviours by PLWH can be shared across different people, institutions and practices. For example, a healthcare provider or a community practitioner could alert PHUs about a potential nondisclosure of HIV status by a PLWH who is not virally suppressed. However, nondisclosure does not automatically co‐constitute risk. High viral load, as a biological/medical status, is also a key factor in mobilising institutional responses. HIV risk emerges as an effect of interactions among laboratory results, nondisclosure, provider interpretations and reporting protocols rather than a stable phenomenon.

Although provider opinions, nondisclosure and viral load are key considerations, participants further described how sexual partners' complaints play a role when determining risk:

So, like, say, we get a complaint, and then we’re like, okay, is this complaint something that we need to action? So viral load is a huge component of that. So, we’re going to look at either our hospital medical record or [database] to see when was the last time they got a viral load and what was the actual viral load?

(Willow, public health nurse)

This account shows that HIV risk emerges through a complex assemblage that includes data infrastructures, in addition to sexual partner complaints and institutional concerns. Medical records and databases can be used and accessed without patient awareness or consent, exemplifying how viral load serves as a technology of surveillance through which public health can act on PLWH without direct interaction.

The enactment of public health risk within HIV risk assemblages is a product of the dynamic interplay among viral load, as determined by laboratory results, providers, public health personnel, clinical judgement, legislation and institutional practices, which positions whether a PLWH is a potential transmission risk. Although factors such as sexual behaviour, intravenous drug use, expert assessments and nondisclosure are considered, viral load in the age of U = U operates as a significant force. The determination of viral load status is based on consultation of medical records and databases, highlighting the constitutive entanglement of human and nonhuman actors. In this way, HIV risk is actively produced through socio‐technical assemblages.

### 3.2 Care Infrastructures

Care infrastructures also emerged as significant processes in the enactment of risk. Many participants underscored the importance of the care cascade, especially care linkage, ART initiation for newly diagnosed people and adherence to medication for those already diagnosed, as factors contributing to PLWH becoming risks. The care cascade itself is made up of heterogeneous relations of humans (including patients, sexual partners, clinicians and public health authorities) and nonhumans (including ARVs, prescriptions, viral load and medical records, including pharmacy records) that are drawn together in an HIV risk assemblage. For example, one participant described:

So definitely like new diagnoses that are not well attached to HIV care […] and people who either know that they’re not taking ARVs or they’re really routinely declining blood work and public health knows that they’re having like many sexual contacts.

(Jordan, public health nurse)

Engagement in care, medication adherence and routine bloodwork are articulated together in the configuration of risk. Other elements, including viral load information, sexual practices and HIV status disclosure, are also entangled.

Normative expectations that PLWH engage in care and follow the care cascade, along with safer sex and disclosure protocols, contribute to the configuration of risk. In addition, practitioners' concerns about exposing others to HIV also form a key element. For example, one interviewee shared:

Sometimes we will get […] a report from a physician […]. One of the patients has not been adherent or compliant with therapy, and that there is a risk that they are spreading the infection […]. We only learn of them when there is a reason to believe that they might be putting others at risk

(Reese, public health nurse)

Different forces, including physicians, medications, patient behaviours and public health legislation, coalesce into an HIV risk assemblage. Various information mechanisms are also articulated into this network. For example, a participant described checking prescription renewal to determine if a person is adherent, but has failed to engage in regular bloodwork to determine risk:

We can also sometimes see if someone is filling their prescriptions for antiretrovirals […]. So, that gives us some information, too, if someone is on treatment but, for whatever reason, isn’t doing regular blood work. So, not to say that they aren’t in care, right? It’s kind of helpful to have some different pieces of information. And we may follow up with their care provider, too, if we can’t find that information ourselves […].

(Micah, public health nurse)

Enacting someone as a risk in relation to the HIV care continuum involves a form of detective work for public health actors, termed ‘sleuthing’ by some participants, where they put together pieces of information across systems and sites. This process involves various practices, expertise and objects, including prescriptions, pharmacies, medications, pharmacists and clinicians, highlighting the relationality of HIV risk assemblages. These elements are used when co‐constituting HIV risk. Therefore, HIV risk is a consequence of the shifting configuration of forces within assemblages.

However, instead of admonishing PLWH for being out of care, public health actors reported using strategies to reintegrate them into the care cascade, including addressing barriers. One participant noted:

We always want to work with people without sort of heading towards Section 22 immediately. […]. It’s really about trying to see what the barriers of care are, what are the barriers to adherence to taking their meds.

(Saga, public health policy/surveillance)

Rather than immediately issuing public health orders, public health authorities prioritise working with patients to re‐establish connection to the care cascade. This involves mobilising various approaches, including communicating with patients, providing incentives and collaborating with their providers to address barriers to HIV care. This suggests that service access is also enmeshed in the materialisation of HIV risk, which is not only mitigated through coercive interventions but also involves reintegration into care. Although some of the practices to improve care engagement described by participants, such as providing incentives, could be rendered supportive and promote health equity, they may also be considered as mechanisms through which public health enacts discipline by reintegrating PLWH into biomedical and surveillance systems (Guta et al. 2016).

### 3.3 STBBI Co‐Infection

The identification of an STBBI co‐infection in a PLWH also emerged as an important element in HIV risk assemblages. STBBI co‐infection activates specific practices among public health personnel, including sleuthing, drawing on various informational systems and data in the shaping of HIV risk. Participants reported that the identification of a co‐infection triggered them to consult patient records to determine viral load and if the patient had potentially exposed someone to HIV transmission. For example, one participant said: ‘So at the time that the viral loads become really important to our work is when someone has been diagnosed with another bacterial STI infection’ (Micah, public health nurse). Another interviewee articulated:

When we’re handling clients with HIV who also might have gonorrhoea, chlamydia, or syphilis, some of our follow‐up is based on their viral load and when the timing of that viral load is […]. Because that would indicate if they could possibly have exposed someone to HIV, it also kind of illustrates if they’re in any kind of care. And we might also even be able to figure out what medication they’re taking.

(Quinn, public health nurse)

Here, viral load emerges as a diagnostic tool, an information system and a mechanism for risk control. By drawing on medical records, institutional expertise and knowledge of sexual behaviours, public health practitioners shape a particular version of risk which, in turn, informs their approach to contact tracing. Specifically, whether to advise partners to test for STBBIs alone or for both HIV and STBBIs. HIV risk emerges through a constellation of STBBI co‐infection, viral load, the timing of that measurement and potential HIV transmission exposure. Public health authorities draw on diverse information infrastructures to inform risk‐determination and partner‐notification strategies for STBBI and/or HIV testing.

Additionally, other participants explained that viral load status determines whether an individual constitutes an ongoing risk and how they should be counselled. If a person is not virally suppressed, they could be subjected to more intense follow‐up, including discussions about dual infections and disclosure responsibilities. For example:

So, let’s say an individual was HIV positive previously and they get a gonorrhoea infection or syphilis infection […]. The next question that we have to figure out is, is this a situation where they are virally suppressed or not […]. And in care? And if they are, then we don’t have to go down any road around dual infection counselling, disclosure responsibility and all those topics.

(River, public health nurse)

STBBI co‐infection, often bacterial and treatable with antibiotics, is a significant element in the configuration of risk. Public health practitioners use various data and infrastructure to determine the intensity of counselling and interventions they should implement. These discussions suggest that STBBI co‐infection, viral load data and public health interventions intertwine in the co‐construction of HIV risk.

### 3.4 Public Health Orders

HIV risk further emerges through complex assemblages of public health legislation, normative apparatus and individual behaviours. Participants described how repeated or ongoing noncompliance with public health directives may configure a PLWH as a risk because they are deviating from expected public health norms and may lead to the issuance of coercive Section 22 orders. For example:

If it kind of happens more than once and starts to become like an ongoing thing […], then public health can issue what are called sections, 22s […] and so on. And that would be the medical officer of health kind of requiring a client to either do or not do particular things. And it can be everything from disclosure, condom use. It can require viral load testing in some situations.

(Quinn, public health nurse)

Noncompliance shifts a PLWH from a client in need of support to someone who must be managed and controlled through a public health order for the safety of others. However, noncompliance itself does not automatically shape a PLWH as a risk. Instead, other elements come together, including nondisclosure, condom use and viral load. Together, these elements reconfigure a PLWH as subversive, perceived as violating public health directives, triggering more coercive measures, which carry the threat of punitive measures through the justice system. As the following participant explained:

If a person is showing that their actions might constitute a risk to others from an infectious disease, we have the power to issue what’s called a Section 22 order […]. And if there is a concern that the individual is not complying with the order, it goes to a justice of peace, which would ultimately make a decision, determination of whether they violate the order and they need to pay a fine.

(Jessie, public health policy/surveillance)

Noncompliance is not simply failure to follow instructions but an event that activates public health's capacity to regulate behaviours. Here, risk is relationally produced through the use of legal orders and surveillance mechanisms. However, although public health authorities have the power to issue orders, many participants expressed reluctance to issue them. As Jessie continued: ‘Generally, we're trying to avoid using any specific legal mechanisms to enforce compliance’. Some individuals articulated that issuing a Section 22 was a ‘last resort’ (Charlie, public health administrator), and many described public health orders as ineffective: ‘In my experience, orders aren't particularly effective tools’ (Jordan, public health nurse). These excerpts demonstrate how public health authorities critically engage with section orders, even problematising the usefulness of these mechanisms, in favour of helping clients return to care. Within HIV risk assemblages, legal powers are contested and negotiated, producing different approaches towards PLWH.

### 3.5 U = U Discourses

The adoption of U = U by public health institutions and authorities has significantly reconfigured HIV risk assemblages and what HIV risk means. As evident in the discussions above, whereas discontinuity with care, STBBI co‐infection, HIV nondisclosure and noncompliance to public health orders are significant elements, undetectability or even viral load itself have become powerful forces, informing the actions of public health practitioners. Viral load measurement has reconfigured HIV risk assemblages, leading public health authorities to prioritise STBBI notification or to dismiss nondisclosure complaints. For example:

I think it was 2018 or 2017, one of those years, our medical officer of health signed on to U = U […]. Once she signed on to U = U, that gave us in our programs the liberty to then re‐evaluate our policies and procedures to have them rooted in U = U […]. Since then […], we don’t have to have those discussions about disclosure with our virally suppressed clients anymore […]. It really changed the relationship we have with our clients because they’re more client‐centred.

(Spencer, public health nurse)

U = U has changed public health practices surrounding HIV risk, from a coercive model to a more collaborative approach with patients, provided they are willing to engage in the care cascade. U = U has stabilised public health strategies, which shifted from a directive towards a supportive model focused on retaining PLWH in care and reducing barriers to access:

Sometimes, it can seem very heavy‐handed and very legalistic, right? You know, you need to use a condom […], you need to tell your partner, you need to do this, right? And now, you know, with U = U, we’re able to sort of really dial that back. It becomes less fraught, less tense around lecturing […]. And now, we can really just talk about how we keep them in care, how we remove barriers, how we support them.

(River, public health nurse)

This quote suggests that HIV risk assemblages have mutated with the introduction of U = U, moving public health approaches from prevention‐oriented to a more care‐centred framework focused on improving support systems for PLWH. Despite policy shifts in some PHUs, some participants expressed concerns about the variability in U = U policy adoption in other public health settings. As one participant described:

Some public health units, I think, have since adapted their policies and procedures to consider whether or not people are virally suppressed and to basically enact U = U in their policies and procedures. But there are definitely some public health units who still do not do that. So that would be a huge difference for you if you’re living in [an urban centre] or somewhere else, where their policies and procedures are the same regardless of their viral load.

(Spencer, public health nurse)

This excerpt highlights how the introduction of U = U has produced different responses to HIV risk across public health jurisdictions in Ontario. This variability in HIV risk approaches appears to be modulated by geography, with urban centres implementing more supportive approaches, whereas other PHUs outside urban areas may apply harsher public health interventions. These different approaches suggest that HIV risk assemblages shift with configurations of U = U policies and urban/nonurban infrastructures. Although U = U has not entirely eliminated risk, it has nonetheless transformed how some PLWH are deemed risky, compliant or manageable. However, it must be noted that several structural barriers continue to affect PLWH's capacity to achieve and maintain undetectability, including financial and housing insecurity, stigma, racial discrimination, criminalisation of HIV and immigration status (Odhiambo et al. 2023), which are entangled in the materialisation of HIV risk.

## 4 Discussion

Our analysis demonstrates that HIV risk is enacted through an assemblage of viral load monitoring, care infrastructures, STBBI co‐infections, public health orders and U = U discourses. Within this assemblage, undetectable viral load functions as an important technology organising the reflexive governance of HIV risk. Although public health has the authority to issue and enforce orders to PLWH if they pose a risk to others (Department of Justice Canada 2017), our findings demonstrate that some public health personnel and units in Ontario have generally avoided issuing these orders immediately, if at all, especially when an undetectable viral load is confirmed. Drawing on Deleuzeoguattarian assemblages framework has helped us demonstrate that viral load measurement has reconfigured how HIV risk is enacted and approached by various public health actors.

Although the tendency against moving quickly to issue orders is laudable, policies ought to guide a reasoned and evidence‐based escalation of interventions from least to most intrusive, with public health orders as the most intrusive and should only be deployed as a last resort. Any decision to issue an order should never be punitive but should be based on clear evidence that all other measures have been ineffective. Such policies should be consistent across PHUs with centralised guidance from Public Health Ontario (PHO) and made publicly available. Transparency and predictability are vital to an ethical and human rights‐respecting approach to coercive interventions and to engendering public trust. In addition, the use of these orders ought to be studied and evaluated for effectiveness, given the coercive impact and intrusiveness on PLWH subject to them. PHO should reinstate public reporting on the issuance of such orders and engage PLWH in studies about the utility of such orders. PHO should also assess the impact and efficacy of required actions to ensure that no measures found to be ineffective are ordered.

Viral load measurement emerged as a privileged force in the co‐shaping of HIV risk. Our findings suggest that a higher viral load does not automatically make someone a transmission risk. Instead, a high viral load operates relationally, becoming significant only when accompanied by activities carrying potential HIV risk, including engaging in sex without using prophylaxis, co‐infection with STBBIs, sharing injection equipment or nondisclosure. Conversely, an undetectable viral load neutralised risk, even when a PLWH engaged in what might otherwise be considered as high HIV transmission activities. This suggests that viral load, alongside other factors such as behaviour, helps determine how risk is interpreted and mobilised.

Another element in the enactment of HIV risk is care infrastructures, linked to the continuum of care or care cascade. This is characterised by a sequence of medical care stages that PLWH must complete, often conceived of as linear (Kay et al. 2016). Some participants described nonengagement with the care cascade as a potential risk indicator. However, previous studies have suggested that many PLWH experience the continuum less straightforwardly. They can skip steps, regress, exit the continuum or return to care again (Kay et al. 2016). When PLWH are not linked to care, participants described drawing on different sources, including prescription renewals and provider follow‐ups, to obtain their care status and viral load results—a practice known as sleuthing. This indicates that various experts and objects are consulted to assess risk. Sleuthing, however, is often done without PLWH's knowledge or consent. This lack of consent is concerning, as it introduces the possibility of increased surveillance and could lead to a reduction in rights to privacy and bodily autonomy, or even more coercive public health interventions. Although such access is permitted under existing public health legislation (Personal Health Information Protection Act 2004), the use of this information raises concerns about individuals' right to consent and the possible use of such data for criminalisation. Therefore, accountability and transparency mechanisms should be in place to ensure that the use of medical records and viral load data does not lead to unnecessary surveillance and further stigmatisation of PLWH. These mechanisms may include, for example, PLWH knowing when and how their records are being accessed.

Notably, instead of admonishing PLWH for regressing, exiting or skipping steps in the care continuum, many public health practitioners indicated applying a more equity‐informed approach to health. However, we argue that an equity‐based approach should involve collaboration with PLWH and also address systemic barriers, including housing and financial insecurities, racial discrimination, HIV criminalisation, immigration status and stigma (Odhiambo et al. 2023). These equity‐informed approaches to HIV care represent a response that closely aligns with Ontario's HIV Action Plan to 2030, which advocates for a holistic approach to addressing HIV vulnerability (Giliauskas 2025).

STBBI co‐infection and noncompliance with public health directives are also assembled in the enactment of HIV risk. However, each of these elements becomes significant only when accompanied by a high viral load. On the one hand, PLWH who are not virally suppressed could be configured as a high transmission risk and subjected to intensified counselling, including discussions about co‐infection and disclosure responsibilities. On the other hand, PLWH who are noncompliant with public health directives, do not conform to safer sex norms and have a high viral load may be approached as someone needing control. Some public health practitioners stated that compliance orders may be escalated, potentially leading to fines. This suggests that legal apparatus and public health norms are entangled within HIV risk assemblages, with the possibility of punitive measures if a person does not align with the public health expectations.

Considering HIV transmission risk as emergent through assemblages, rather than simply fixed or a given category, enabled us to surface the dynamic role of viral load measurement and other interconnected elements in reconfiguring how public health co‐constitutes risk in light of U = U. As many participants highlighted, adopting U = U in their local PHUs has reshaped their practice when counselling PLWH. Within these PHUs, practitioners described that discussions about disclosure and other obligations have been largely disregarded for virally suppressed clients, suggesting that some public health actors reflexively interpret risk in relation to viral load measurement. This implies that viral load is a significant technology in the governance of risk. However, some participants noted that adopting U = U is inconsistent in Ontario, as some PHUs continue to practice severe interventions when engaging with PLWH despite viral suppression. This uneven acceptance of U = U across all PHUs in Ontario is troubling, given PHO's consistent messaging that virally suppressed PLWH pose a negligible risk of transmission (Ontario Ministry of Health 2023). PHO must ensure that each local PHU integrates U = U into its practice and decision‐making and produce standardised decision criteria when determining risk. It is also vital that all PHUs adopt approaches that prevent the use of punitive measures.

This study contributes to the growing literature on HIV assemblages (Brown and Di Feliciantonio 2022; Guta et al. 2016; McClelland 2019; Odger et al. 2019; Rinaldi and Marques 2020), with a specific focus on HIV risk assemblages. It also contributes to research focussing on how PLWH's behaviours are considered HIV transmission risks by public health practitioners within the context of U = U and the care cascade in Canada (e.g., Mykhalovskiy 2014; Newman et al. 2018). To the best of our knowledge, our study is one of the few that draws specifically on Deleuzeoguattarian assemblages framework to analyse interview data from public health practitioners regarding their views on PLWH's behaviours as transmission risks, enabling us to demonstrate that PLWH are actively co‐constituted as risky through relational practices encompassing various fields of power, knowledge and social interactions. More importantly, our study finds that an undetectable viral load almost always neutralises risk even when other factors are present. This highlights how viral load measurement operates as a technology of reflexive governance, allowing public health to disregard other risk indicators completely.

This study enriches assemblage theoretical approaches to HIV research by demonstrating how HIV risk emerges through different relations, whilst highlighting the critical and reflexive role of public health practitioners in enacting risk. More broadly, we contribute to operationalisations of risk in the sociology of health and illness, moving away from individualised and behavioural notions towards a relational and more‐than‐human approach that attends to how risk is produced and governed. Health conditions emerge as public health threats through specific configurations of human/nonhuman forces and need to be examined as the effects of intersecting legal, economic, racialised, gendered and biomedical arrangements, not simply as results of individual conduct.

This study has several limitations. First, it was conducted with a focus on Ontario, limiting the transferability of the findings to other Canadian jurisdictions where public health governance, legal interpretations, and the uptake of U = U may differ. Comparative research across provinces and territories is needed to explore how local law, medicine and community assemblages shape HIV risk discourses. Second, the perspectives presented here are primarily from professionals working within larger, mostly urban PHUs and may not reflect practices in smaller or rural settings. Further studies should account for local variations. Finally, whilst our findings hint at the entanglement of public health and criminal law, we did not thoroughly examine how these interlocking systems regulate PLWH. However, members of our team have done extensive work on the impacts of HIV criminalisation (Hastings et al. 2024). Future research should critically attend to this intersection, especially in relation to equity and the lived experiences of PLWH and other marginalised groups.

Viral load and U = U have become influential in guiding more supportive, noncoercive and nonpunitive public health responses. However, obtaining viral load information to determine risk is often done without PLWH's knowledge or consent. Accessing viral load information should be restricted only to situations where clear evidence of current or ongoing behaviours considered HIV transmission activities exists. Additionally, centralised guidance about U = U and nonpunitive practices must be established to ensure consistency across all PHUs. Transparency about the issuance of Section 22 orders is also needed. A more consistent, transparent and accountable approach would promote more equitable and nonstigmatising practices to HIV risk across the province.

## Author Contributions

**Emerich Daroya:** conceptualization, funding acquisition, writing – original draft, validation, writing – review and editing, formal analysis, data curation. **Martin French:** conceptualization, funding acquisition, writing – review and editing, validation, formal analysis, data curation. **Colin Hastings:** conceptualization, funding acquisition, writing – review and editing, validation, formal analysis, data curation. **Andrea Krusi:** conceptualization, funding acquisition, writing – review and editing, validation, formal analysis, data curation. **Stephen Molldrem:** conceptualization, funding acquisition, validation, writing – review and editing, formal analysis, data curation. **Maureen Owino:** conceptualization, funding acquisition, validation, writing – review and editing, formal analysis, data curation. **Ryan Peck:** conceptualization, funding acquisition, validation, writing – review and editing, formal analysis, data curation. **Amy Wah:** conceptualization, funding acquisition, validation, writing – review and editing, formal analysis, data curation. **Alexander McClelland:** conceptualization, investigation, funding acquisition, methodology, validation, writing – review and editing, formal analysis, project administration, resources, data curation, supervision.

## Funding

This work was supported by the Canadian Institutes of Health Research (Grant No. 467746).

## Data Availability

The qualitative interview data supporting this article are not publicly available in order to protect the privacy and confidentiality of participants, as outlined in the approved ethics protocols.
